# Genomic basis of scent loss and functional divergence in wild and cultivated carnations (*Dianthus* spp.)

**DOI:** 10.1093/hr/uhag130

**Published:** 2026-04-07

**Authors:** Jesús Picazo-Aragonés, Stefan Dötterl, Ovidiu Paun, Anass Terrab, Francisco Balao

**Affiliations:** Department of Plant Biology and Ecology, University of Seville, Seville, Spain; Department of Environment and Biodiversity, University of Salzburg, Salzburg, Austria; Department of Botany and Biodiversity Research, University of Vienna, Vienna, Austria; Department of Plant Biology and Ecology, University of Seville, Seville, Spain; Department of Plant Biology and Ecology, University of Seville, Seville, Spain

## Abstract

Domestication reshapes plant genomes and traits, yet the genomic basis of floral scent loss remains unclear. Here, we present an 876 Mb pseudo-chromosome genome assembly of the wild carnation *Dianthus broteri* (2*n* = 2*x* = 30) and compare it with the cultivated *D. caryophyllus*, which exhibits reduced floral scent. Transposable elements occupy 82.4% of the *D. broteri* genome, dominated by Gypsy LTRs, whereas *D. caryophyllus* exhibits more recent insertions near genes, consistent with domestication associated effects. Volatilome profiling of *D. broteri* revealed 59 floral volatiles, dominated by sesquiterpenoids, with lineage-specific differences in composition. Terpenoid-biosynthesis genes are upregulated in flowers of the scented western lineage, while the weakly scented eastern lineage shows reduced expression of upstream pathway components despite constitutive transcription of some terpene synthases. Variant discovery within terpenoid pathway genes showed that the most highly and constitutively expressed gene, *DbrTPS18,* accounted for almost 50% of all lineage-specific variants. Several nonsynonymous substitutions in *DbrTPS18* were identified within the catalytic domains and the predicted chloroplast transit peptide, and these variants were significantly associated with variation in scent production. These results uncover the basis of terpenoid biosynthesis in the genus *Dianthus* and, together with the presented reference genome, provides a foundation to explore the genomic consequences of domestication and enhance fragrance in cultivated carnations.

## Introduction

Domestication represents one of the most transformative processes in human history, reshaping wild plants and animals into species that sustain societies [1, 2]. Beginning ~10 000 years ago, artificial selection for traits of interest enabled the transition from hunting-gathering to farming, supporting human population growth and the rise of complex civilizations [3]. While domestication has yielded tremendous benefits [4], it has also introduced genetic costs, such as reduced diversity, accumulation of deleterious variants, and erosion of adaptive traits [4–6]. Understanding the genomic consequences of domestication remains of central interest in evolutionary biology and applied breeding.

The advent of high-quality chromosome-level genome assemblies has revolutionized plant genomics, allowing researchers to detect structural variants such as changes in gene content or transposable element (TE) activity. These findings can be related with the evolution of traits of interest, including agronomic traits in domesticated species, due to gene family expansion, regulation of the gene expression, and gain/loss of gene functions [4, 7, 8]. However, high-quality genomes of crops’ close wild relatives are still scarce, limiting our ability to trace the genomic consequences of domestication and harness wild diversity for breeding.

Cultivated carnations (*Dianthus caryophyllus* L.) are among the most important ornamental crops worldwide, cultivated for >2000 years and valued for their wide range of flower colors, shapes, and long vase life [9]. *Dianthus* (Caryophyllaceae) is a highly diverse Eurasian genus, centered in the Mediterranean Basin, with >300 carnations and pinks species, and is notable for high incidence of polyploidy and the fastest diversification rate in flowering plants [10, 11]. Carnation domestication is believed to have originated in the Mediterranean region, especially around Italy and Greece, and involved both intra- and interspecific crossings resulting in a tremendous number of varieties over time [12–14]. While multiple genomes of cultivated varieties have been published in the last decade [9, 14–16], genomic resources for wild relatives remain largely absent. This is a critical gap, as wild *Dianthus* species harbor traits such as drought tolerance and strong fragrance, which are largely diminished in cultivated carnations due to historical focus on aesthetic traits over ecological ones [17].

Floral scents, ecologically important secondary metabolites that mediate plant–pollinator interactions, defense, and interplant signaling, among other ecological functions [18, 19], are a striking example of trait erosion through carnation domestication. Over 1700 volatile organic compounds (VOCs) are so far known [19, 20], broadly classified into three major groups: phenylpropanoids/benzenoids, terpenoids, and fatty acid derivatives (FADs) [21]. While floral scent chemistry and fragrance strength strongly varies among wild *Dianthus* species, with overall compounds of all these three major chemical groups available in the genus [22], most cultivated carnations typically emit only a faint fragrance dominated by benzenoids, especially methyl benzoate and eugenol [17, 23]. In this regard, wild *Dianthus* species are thus an untapped reservoir of fragrance-related genes that could be used to restore scent diversity in modern cultivars [17].

Here, we present the first high-quality genome assembly of a wild carnation, the diploid cytotype of *Dianthus broteri* Boiss. & Reut. [24]*. D. broteri* complex, distributed across the Iberian Peninsula, has been a model for studying polyploidy, adaptation and volatile-mediated pollinator interactions [25]. It is a perennial herb adapted to dryness [10, 26] with flowers rich in scent that shape their pollinator spectra, exhibiting different volatile profiles among cytotypes [27]. In this research, we focus on the diploid cytotype that includes two disjunct diploid lineages with separate origin, identified with genetic methods in a population-level study [24], inhabiting the mountain ranges in the south of Portugal and Spain. Here, we combine comparative genomics between wild carnations (*D. broteri* and *D. sylvestris*) and cultivated varieties ‘Aili’ and ‘Francesco’ of *D. caryophyllu*s, with volatilome profiling and VOC biosynthetic pathway analyses to shed light on how domestication reshaped genome structure and scent diversity. Our findings provide valuable genomic resources, including candidate genes, for breeding programs aimed at improving ornamental traits, particularly fragrance and drought resilience, in cultivated carnations.

## Results

### A high-quality genome assembly of *Dianthus broteri*

We generated a chromosome-level reference genome for the diploid cytotype of *D. broteri* (2*n* = 2*x* = 30) using PacBio CLR reads (126 Gb, ~143× coverage) and Illumina data (51 Gb, ~58× coverage). The genome size of *D. broteri* was estimated to be 885.5 Mb by flow cytometry (FCM), whereas K-mer analysis (*K* = 21) reported a genome size of 776 Mb. Relatively high heterozygosity (1.04%) and high repetitiveness (84%), which compounds the heterozygosity effect, likely contribute to an underestimation of genome size due to misinterpretation of K-mer frequency distributions [28]. Given the limitations of K-mer analyses for complex and repetitive plant genomes, and considering previous genome size estimates for *D. broteri* obtained by FCM [29], we considered FCM value to more accurately reflect the true nuclear DNA content. The initial haploid assembly spanned 1.09 Gb over 731 contigs (N50 = 10.99 Mb). After haplotig purging, polishing and homology-based scaffolding using *D. caryophyllus* ‘Aili’ [9] as reference, we obtained a final assembly of 876 Mb, consistent with estimated haploid genome size, with a GC content of 37.1%, N50 of 53 Mb and 34 scaffolds. Among these, 15 corresponded to pseudo-chromosomes, representing >86% of the estimated genome size, while the remaining 19 were minor scaffolds.

Completeness and accuracy of the resulting genome assembly were assessed using multiple complementary approaches. Completeness was examined with Benchmarking Universal Single-Copy Orthologs (BUSCO), which indicated that 94.5% of the core eudicotyledons genes were present in the *D. broteri* genome assembly, of which 93.4% had complete coverage (76.8% were single copy and 16.6% were duplicated). Furthermore, base-level accuracy and completeness were evaluated analyzing the K-mer spectrum of Illumina reads with Merqury [30], resulting in a quality value (QV) of 46.7 (base-level error rate of ~2.1 × 10^−5^). Additionally, long terminal repeat (LTR) Assembly Index (LAI) of 12.12 categorized *D. broteri* genome as a reference genome (10 ≤ LAI < 20). Standarized repeat annotation and LTR detection pipeline across all *Dianthus* assemblies showed that *D. broteri* presented similar LAI as *D. caryophyllus* ‘Aili’ (11.98), and higher LAI than ‘Francesco’ (0.26) and ‘Scarlet Queen’ (2.98) genomes. Finally, DNA and RNA mapping results also demonstrated the high quality of the produced genome assembly, with 99.16% of DNA mapped reads (of which 98.84% were correctly paired) and 100% RNA mapped reads (86% uniquely mapped).

Structural annotation, using a *de novo* assembled transcriptome and annotated repetitive elements, together with protein sequences as evidence (Material and Methods, Structural and functional gene annotation), resulted in 43 817 gene models, with an average gene length of 3613.2 bp and five exons per gene (Table 1). Gene content in *D. broteri* was practically the same as in the cultivated *Dianthus* genomes [9, 14–16]. Annotation completeness was evaluated using eudicots_odb10 database in BUSCO, showing a score of 92.9%.

**Table 1:** Genome assembly contiguity and annotation statistics between *Dianthus* species

**Genome attribute**	***D. broteri***	***D. caryophyllus* ‘Aili’**	***D. caryophyllus* ‘Francesco’**	***D. sylvestris***
Length	876.18 Mb	582.39 Mb	568.89 Mb	443.52 Mb
*n* Scaffold	34	15	45 088	21 369
N50	539.1 Mb	374 Mb	60.7 Kb	60.5 Kb
L50	7	8	2082	2026
*n* Genes	43 817	44 098	43 266	21 915
Mean *n* exons	5	4.2		
Mean *n* introns	4	3.2		
Mean gene length	3613.15	3156.5	2737.31	2927.32
Median exon length	234	280		
Median intron length	646	620		
TE Length	722.16 Mb	403.8 Mb	306.97 Mb	142.41 Mb
TE %	82.42%	69.33%	53.96%	32.11%
BUSCO	94.5	97.15	94.5	89.2

### Transposable element burst in *Dianthus broteri* compared with domesticated relatives

**Figure 1:**
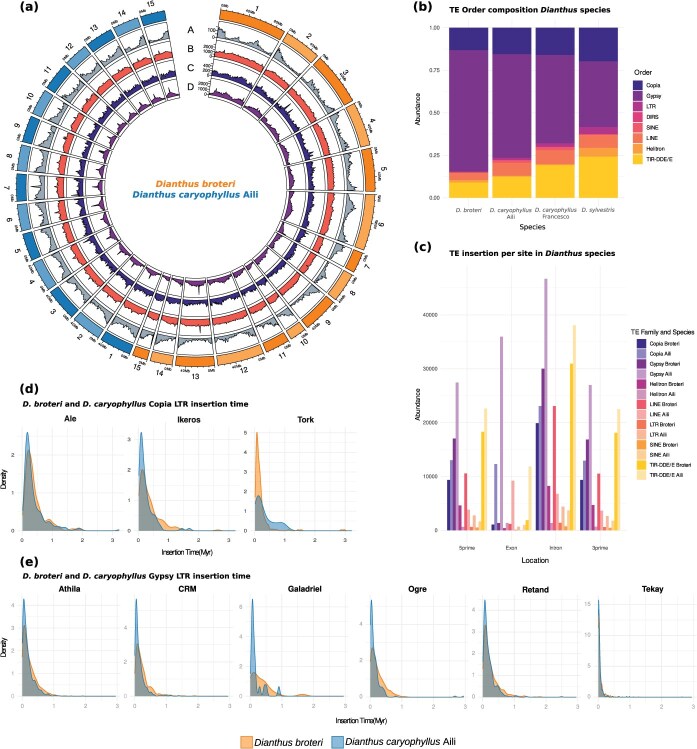
Transposable element composition and dynamics across *Dianthus* species. (a) Circular plot representing 15 chromosomes of *D. caryophyllus* ‘Aili’ and 15 pseudo-chromosomes of *D. broteri*. Going inward, tracks represent gene (A), global transposable elements (B), Copia LTR (C), and Gypsy LTR (D) density across the genomes with a window size of 1 Mb. (b) Relative TE composition comparison between wild (*D. broteri* and *D. sylvestris*) and cultivated (*D. caryophyllus* ‘Aili’ and ‘Francesco’) carnations. The graph shows a significant expansion of Gypsy elements in wild *D. broteri* (Chi square test *P*-value <0.05). (c) Per site TE insertion analysis in wild *D. broteri* and cultivated *D. caryophyllus* ‘Aili’ genomes. (d) Insertion time LTR Copia and (e) LTR Gypsy subfamilies that are significantly different inserted between *D. broteri* and *D. caryophyllus* ‘Aili’.

*D. broteri* genome contained a total of 722.1 Mb of repetitive elements (2 015 482 repeats, 1 145 290 being complex repeats/TEs) as identified by RepeatMasker, representing 82.42% of the total genome size (Table 1). The predominant TEs were, by far, LTRs, accounting for 60.55% of the total genome length. Within LTRs, Gypsy elements were the most abundant (50.77%), followed by Copia elements (9.41%). Other important TEs in the *D. broteri* genome were DNA TEs (TIR-DDE/E, 6.41%), LINE L1 (2.51%), and Helitron (1.08%) (Table S1). Compared with other *Dianthus* species, we observed an expansion of TEs in *D. broteri*. For instance, in the *D. caryophyllus* var. ‘Aili’ and ‘Francesco’, TEs constituted 69.33% (403.8 Mb) and 53.96% (306.9 Mb) of their genomes, respectively, and for *D. sylvestris* TE content was much lower, representing only 32.11% (142.4 Mb). These differences in the content of repetitive elements were correlated with differences in genome size between *Dianthus* species, which was explained by the significant expansion of Gypsy LTRs in *D. broteri* (χ^2^ test *P*-value <0.001, Fig. 1b).

We studied differences in insertion time between cultivated and wild carnations to investigate the evolutionary role of LTR retrotransposons (LTR-RTs). Results revealed that the Gypsy superfamily insertion was older than Copia in both *D. broteri* and *D. caryophyllus* ‘Aili’ (*D. caryophyllus* ‘Francesco’ and *D. sylvestris* were excluded from this analysis due to genome fragmentation). Comparison between species showed that Gypsy elements insertions are more recent in *D. caryophyllus* (Wilcoxon test, *W* = 4 981 434; *P*-value <0.001), peaking at 92.3 thousand years ago (Kya), while in *D. broteri* insertions peak at 136.04 Kya. However, Copia superfamily showed a similar insertion peak for both cultivated (147.17 Kya) and wild (143.34 Kya) *Dianthus*. The insertion times for specific Copia and Gypsy lineages also exhibited differences between the species. For Copia lineages, Ale and Ikeros experienced older bursts in *D. broteri*, contrasting with the Tork lineage, which showed a more recent and explosive expansion in *D. caryophyllus* ‘Aili’ (Fig. 1d). A similar contrasting pattern was observed in Gypsy lineages, where only Galadriel exhibited a more recent burst in wild carnations (*D. broteri*, Fig. 1e). A detailed summary of the insertion time peaks for all Copia and Gypsy lineages is provided in Table S2. Interestingly, the target sites for TE insertions differed between wild and cultivated *Dianthus* (Fig. 1c). Despite the pronounced transpositional activity in *D. broteri*, *D. caryophyllus* ‘Aili’ exhibited a higher insertion rate of TEs in genic regions (exons and introns) and within 1-kb regions upstream and downstream of genes. This trend was consistent across most TE families, except for Helitron and LINE elements, which had a higher insertion rate in gene-proximal and intronic regions of *D. broteri*.

### Gene family dynamics between wild and cultivated *Dianthus*

**Figure 2:**
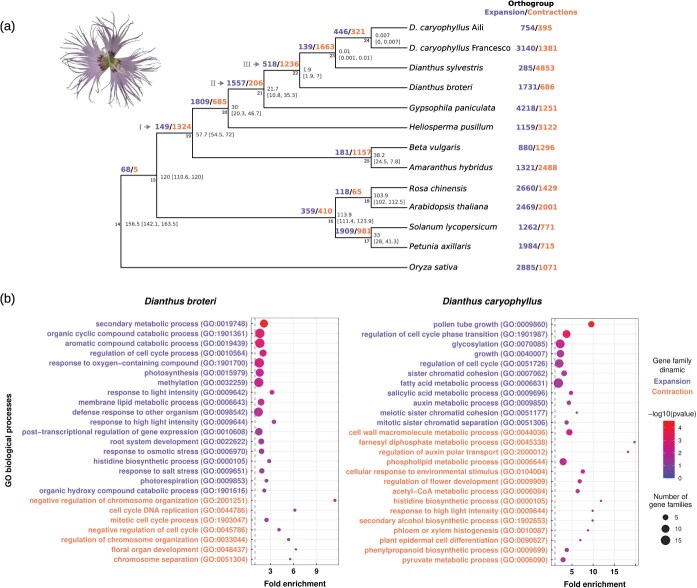
Gene family dynamics in *Dianthus.* (a) Ultrametric phylogenetic tree based on single-copy orthogroups from 13 plant species of which *D. caryophyllus* ‘Aili’ and ‘Francesco’ represent cultivated *Dianthus,* and *D. broteri* and *D. sylvestris* wild ones. Gray arrows I, II, and III indicate ancestral node for Caryophyllales order, Caryophyllaceae family, and *Dianthus* genus, respectively. *Oryza sativa* has been included as an outgroup. The numbers on top of the nodes indicate expansion (purple-blue) and contraction (orange) of gene families. Gray numbers in the nodes indicate diversification time, with confidence interval in brackets. Node calibration times were obtained from TimeTree (Table S3) Picture on top is *D. broteri* 2x flower. (b) GO enrichment analysis of expanded (purple-blue) and contracted (orange) gene families in last common ancestor of *D. broteri* and *D. caryophyllus*. Circles size represents the number of gene families associated to each enriched GO term and the color the significance of enrichment in logarithmic scale (red – more significance, blue – less significance).

To explore the dynamics of gene families in *Dianthus*, including genes of the terpenoid biosynthetic pathway, we compared the available genomes from the *Dianthus* genus together with other nine species along angiosperm phylogeny: *Amaranthus hybridus*, *Arabidopsis thaliana*, *Beta vulgaris*, *Gypsophila paniculata*, *Heliosperma pusillum*, *Oryza sativa*, *Petunia axillaris*, *Rosa chinensis*, and *Solanum lycopersicum*. Genes from the 13 species (including the two cultivated and two wild *Dianthus*) were clustered into 33 720 orthologous groups (OGs). Our phylogenetic tree of species resulted in similar branching events compared to previous studies [31]. We spotted significant gene expansion in the nodes representing the diversification of Caryophyllaceae (Fig. 2a), which can be explained by the ancestral whole-genome triplication of the family [31, 32], and a significant contraction in the crown node of the *Dianthus* genus and the most recent common ancestor (MRCA) between wild and cultivated carnations.

Gene Ontology (GO) enrichment analysis between gene families of *D. broteri* and the cultivated *D. caryophyllus* MRCA allowed us to investigate which gene functions were affected by these expansions/contractions. In wild *D. broteri*, our results revealed a significant expansion of gene families associated with secondary metabolic processes, organic compound biosynthesis, and multiple stress response mechanisms (Fig. 2b). In contrast, many of these processes were not only absent but contracted in cultivated carnations. Notably, among the contracted gene families in cultivated carnations were those involved in flower development and the biosynthesis of volatile organic compounds, such as farnesyl diphosphate, phenylpropanoids, and acetyl-CoA metabolic pathways. Gene families associated with development and growth showed significant expansion in cultivated carnations (Fig. 2b).

### Floral scent diversity in the diploid *Dianthus broteri*

**Figure 3:**
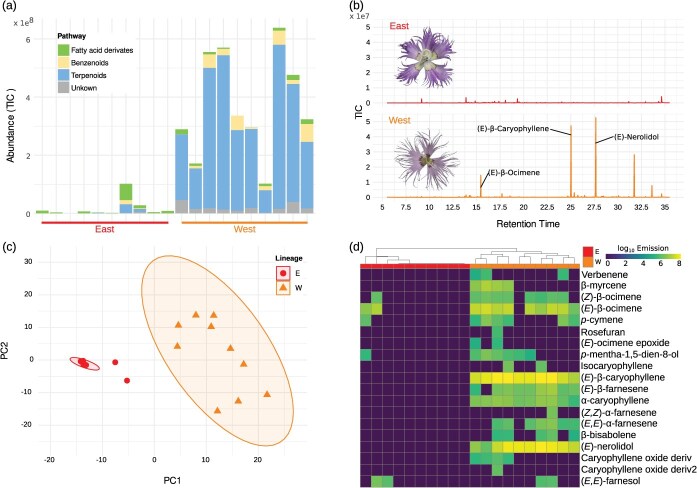
Volatilome comparison between *D. broteri* West and East lineages*.* (a) Absolute emission of VOCs in 10 samples from the East and West lineages. (b) Examples of GC-MS profiles of West and East lineages, with the top three emitted terpenoids indicated. (c) PCA of the West and East samples based on the relative amounts of the compounds displaying 95% concentration ellipses. (d) Heatmap of relative terpenoid abundance (log 10). Samples are hierarchically grouped by location.

Compared to *D. caryophyllus* volatilome*,* primarily composed of benzenoid methyl benzoate and eugenol [33], *D. broteri* presents a much richer floral scent. We characterized the scent profile of wild 2x *D. broteri* by sampling the VOCs from 20 flowers (one per individual), 10 each from the two independent lineages of this cytotype (East and West, Table S4) [24, 29]*.* Gas chromatography coupled with mass spectrometry (GC/MS) analysis revealed a total of 59 compounds characterizing *D. broteri* volatilome predominated by terpenoids. A Kruskal–Wallis test and a permutational multivariate analysis of variance (PERMANOVA) showed differences in the total VOC emission (χ^2^ = 37.55, *P*-value <0.001) and the chemical composition (pseudoF = 14.61, *Df* = 1, *R*^2^ = 0.462, *P*-value <0.001; Fig. 3) of the two phylogeographical lineages. Eastern populations exhibited weak or nearly undetectable floral scents, with a total of 21 scents present in the scent profile, characterized by the fatty acids 2-tridecanone (26.1% of the total emissions in East compared to 1.09% of the total emission in both lineages) and (*Z*)-3-hexenyl acetate (16.76% compared to 1.79%), the monoterpenoid (*E*)-β-ocimene (19.93% compared to 9.03%) and the phenylpropanoid benzyl benzoate (7.94% compared to 2.83%). Opposingly, the western diploid lineage produced a rich volatilome with 52 compounds, dominated by sesquiterpenoids (*E*)-β-caryophyllene (40.4% of the total western volatilome compared to 38.7% of the total 2x cytotype volatilome) and (*E*)-nerolidol (30.08% compared to 28.81%), and the monoterpenoid (*E*)-β-ocimene (8.55% compared to 9.03%) (Table S5).

### Biosynthetic pathways of volatiles in wild and cultivated *Dianthus*

**Figure 4:**
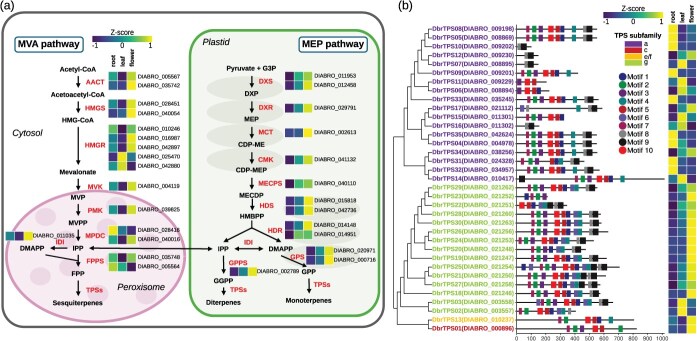
Terpenoid biosynthetic pathway and characterization of terpene synthase-encoding genes in *D. broteri.* (a) Differential expression between tissues (root, leaf, and flower) of the MVA and MEP terpenoid biosynthetic pathways genes characterized using KIPE. (b) Phylogeny of TPS genes identified in *D. broteri* by searching the PF03936 and PF01397 domains with HMMER*.* Subfamily classification of the TPS is based on the maximum likelihood phylogenetic tree build with TPS sequences of *A. thaliana*, *D. broteri*, and *D. caryopyllus* (Fig. S1). Motifs were predicted using MEME. Heatmap represents *z*-scores of the differential expression analysis of the DbrTPS between tissues; genes of the TPS-g, c, and e/f subfamilies show typically greater expression in flowers, while TPS-a subfamily members are mostly overexpressed in root and leaf.

Contrasting with cultivated carnations, wild *D. broteri* presented a much richer scent, characterized by the abundance of terpenoids that are typically synthesized by two different pathways in green plants: the mevalonic acid (MVA) pathway in the cytosol for sesquiterpenoids, and the methylerythritol phosphate (MEP) pathway in plastids for mono- and diterpenoids [21, 34]. Using Knowledge-based Identification of Pathway Enzymes (KIPEs) [35] we identified 16 genes associated with the MVA pathway and 13 with the MPA pathway (Fig. 4a). Additionally, we characterized the terpene synthases (TPS) in *D. broteri*, a midsize gene family that constitutes the terminal enzymes in terpenoid biosynthesis [25]. HMMER [36] identified 35 *D. broteri* TPS genes (DbrTPS) using the two specific domains PF03936 and PF01397. Of these, 28 contained both domains while six candidates retained only the PF03936 motif and just one the PF01397 exclusively. Additionally, comparison with *A. thaliana* TPSs (AthTPSs) using DIAMOND [37] obtained the same result (Fig. 4b). The same approach was replicated in *D. caryophyllus* ‘Aili’, identifying 34 TPS (DcaTPS).

DbrTPS genes were classified into four subfamilies (TPS-a, TPS-c, TPS-g, TPS-e/f; Fig. 4b) using an IQ-TREE maximum likelihood phylogenetic tree [38] with TPS sequences of *A. thaliana* and both *Dianthus* species (Fig. S1). TPS-a and TPS-g accounted for almost 95% of the TPS genes in *D. broteri,* which were found in several clusters along the genome (Fig. S2). Most of the 13 members of the TPS-g subfamily were clustered in pseudo-chromosome six, while the two other members of TPS-g subfamily were in pseudo-chromosome one, in which we also found one TPS-c. Members of TPS-a subfamily present the widest distribution, being present in eight different pseudo-chromosomes, forming two clusters of three and five genes in chromosome three. Moreover, MEME Suite [39] identified 10 conserved protein motifs across the DbrTPS genes (Fig. 4b). While genes in the TPS-g subfamily shared a similar motif structure, those in the TPS-a subfamily displayed greater structural diversity. All the motifs had similar presence across the genes, being the motif six the least common and nine the most. Notably, the TPS-b subfamily was completely absent in both *Dianthus* species.

To understand the role of the genes from the MVA and MEP biosynthetic pathways in the floral scent production, we conducted a differential expression analysis across root, leaf, and flower (petals) tissues (Table S6). In total, 7175 genes were differentially expressed across tissue, with a log2 fold change >1.5 and *P*-value <0.05 after false discovery rate (FDR) correction. Pairwise comparisons showed 18 upregulated and two downregulated genes in flowers from the MVA or MAP biosynthetic pathway (hereafter referred as terpenoids genes) compared with leaf, and 19 upregulated and one downregulated genes compared with root. Differential expression analyses also showed 17 upregulated and four downregulated TPS genes in flowers compared with leaf and 17 up- and six downregulated genes compared with root tissue. These results indicate a general trend of higher expression of genes involved in terpenoid biosynthesis in floral tissues of *D. broteri* (Fig. 4). Notably, the TPS-g genes *DIABRO_021248* and *DIABRO_021246* were among the top 100 most highly expressed genes, which likely produce (*E*)-β-ocimene according to annotation using Contrastive Learning Enabled Enzyme Annotation (CLEAN) [40], one of the major floral volatiles in *D. broteri.*

### Differential expression analysis between scented and unscented *Dianthus broteri* lineages

**Figure 5:**
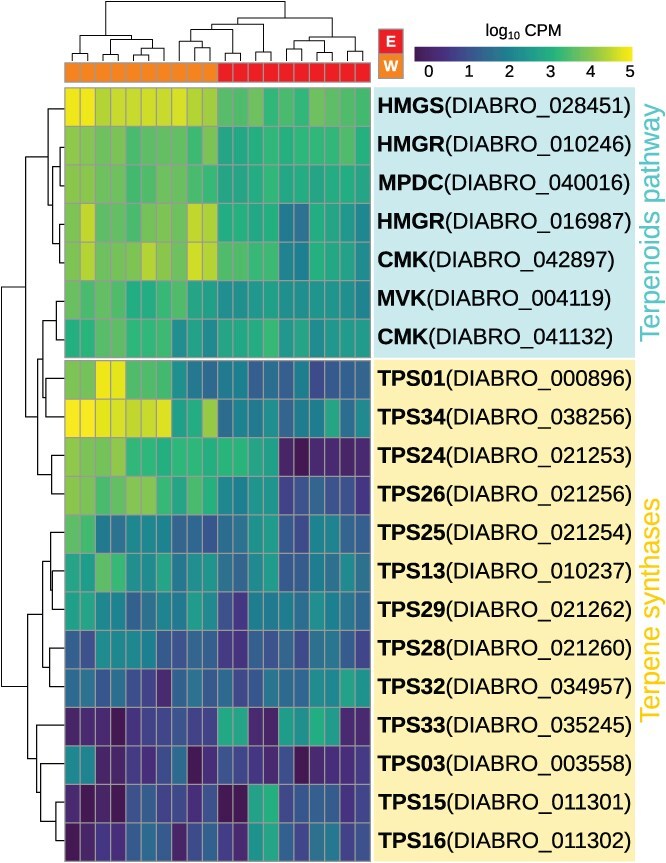
Differentially expressed genes of the terpenoid biosynthetic pathway and TPS genes in East and West *D. broteri* lineages.

To elucidate the mechanisms underlying the differences in scent between the strongly scented western lineage and the weakly scented eastern lineage of *D. broteri*, we studied transcriptomic differences between them. A total of 4925 genes were differentially expressed: among these, nine TPS genes and seven genes from the MVA and MEP biosynthetic pathways were overexpressed in the western lineage, and four TPS genes were overexpressed in the eastern lineage (Fig. 5). Upregulated TPS genes in the East were annotated as members of the TPS-a subfamily, while most upregulated TPSs in the West were members of TPS-g, responsible of the production of (*E*)-β-ocimene and α-farnesene, among other terpenoids corresponding to the CLEAN predictions. Other TPS genes upregulated in the West were one TPS-a, one TPS-c, and one TPS-e/f. Interestingly, some terpene synthases exhibited constitutive expression in both lineages: three TPS-g (*DIABRO_021246*, *DIABRO_021247*, and *DIABRO_021248*, Fig. S3), which products were annotated as (E)-β-ocimene, and one TPS-a (*DIABRO_010417*), which product could not be predicted. This result contrasts volatilome analysis, in which the eastern lineage presents hardly any VOCs.

### Variant calling and association analyses of terpenoid biosynthesis genes

**Figure 6:**
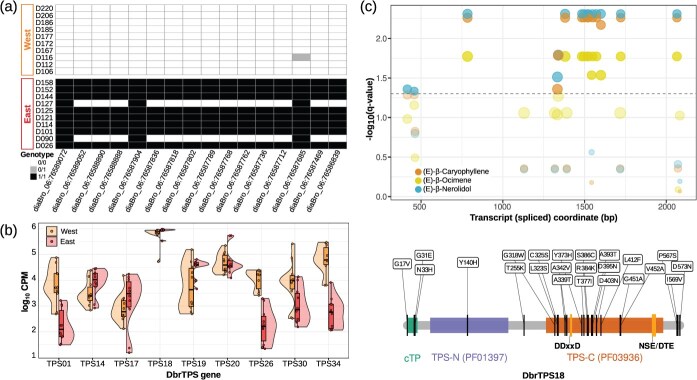
Genetic and functional characterization of lineage-specific variation in the terpene synthase locus *DIABRO_021246* (*DbrTPS18*). (a) Genotype heatmap for lineage-specific SNPs across individuals from eastern and western genetic lineages. (b) Absolute gene expression levels (log_10_ CPM) of TPS genes across samples. Violin plots show expression of western and eastern lineages. *DbrTPS18* shows marked expression relative to other family members. (c) Association results between missense variants in *DbrTPS18* and major floral terpenoids, (E)-β-caryophyllene, (E)-β-ocimene, and (E)-nerolidol. Points show –log₁₀(q-value) along the spliced transcript, and dot size is proportional to effect size estimate. The protein model maps SNP-derived amino acid substitutions across functional regions of the enzyme, including the plastid-targeting peptide and the N- and C-terminal catalytic domains with their conserved active-site motifs.

Variant calling across genes of the terpenoid biosynthetic pathway identified 8741 biallelic single nucleotide polymorphisims (SNPs), of which 495 variants across 31 genes passed quality and genotype filtering following GATK best practices. Most genes showed low to moderate polymorphism (median = 12 SNPs per gene, range 1–51). Among the 39 lineage-specific variants (≥80% within each lineage), *DIABRO_021246* (*DbrTPS18*) was strongly enriched, harboring 18 SNPs and accounting for 46.1% of all lineage-specific polymorphisms detected in the pathway (Fig. 6a; Table S7).

Across the full set of SNPs in *DbrTPS18*, 24 nonsynonymous substitutions were distributed along the coding sequence, clustering within the conserved TPS-C domain. Fifteen substitutions mapped near the canonical DDXXD and NSE/DTE catalytic motifs, and three affected the N-terminal region corresponding to the predicted chloroplast transit peptide (Fig. 6c). ChloroP predictions revealed clear lineage-dependent differences in plastid targeting. A chloroplast transit peptide was predicted in all western individuals, whereas most eastern samples lacked a detectable targeting signal. Single-variant association tests identified 16 SNPs significantly associated with variation in (E)-β-caryophyllene, (E)-β-ocimene, and/or (E)-nerolidol emission (FDR < 0.05), with the strongest associations concentrated in the transit peptide and central and C-terminal regions of the *DbrTPS18* protein (Fig. 6c).

## Discussion

Detecting genomic consequences of domestication can be challenging without high-quality genomes of cultivated species and their wild relatives. In this research, we produced a pseudo-chromosome level assembly of the wild carnation *D. broteri*, with a genome size of 876 Mb. The assembly shows high contiguity and completeness, with 86.56% of the genome anchored to 15 pseudochromosomes. Similarly to cultivated *Dianthus* species [9, 14–16], a total of 43 817 protein-coding genes were annotated, of which 79.26% had predicted functions and 58.39% were assigned at least one GO term. Moreover, with 94.5% of the core eudicotyledons genes present in the *D. broteri* genome, completeness is similar to the cultivated *D. caryophyllus* ‘Francesco’ (94.5%; [16]), ‘Aili’ (97.5%; [9]) and ‘Scarlet Queen’ (97.15%; [14]) genomes. Furthermore, the reference genome has similar LAI to model species such as *A. thaliana* (14.9; [41]), classifying the assembly as a reference genome assembly (10 ≤ LAI < 20). However, when we calculated the LAI across the studied *Dianthus* genomes, we spotted differences from previously reported values for *D. caryophyllus* ‘Aili’ (11.98 compared to 22.13 initially reported [9]) likely due to alternative TE annotation approaches. All the present genomic statistics validate the *D. broteri* genome assembly as the first high-quality assembly of a wild species in the Caryophyllaceae family. As such, it provides a valuable genomic resource for comparative studies between wild and cultivated *Dianthus* species, enabling investigations into the genomic dynamics of domestication and the molecular pathways underlying key traits such as floral scent production [17, 42].

Despite sharing the same chromosome number (2*n* = 30), a major distinction between cultivated *Dianthus* varieties and *D. broteri* lies in genome size, with *D. broteri* exhibiting nearly double the total DNA content compared to its domesticated relatives [9, 14–16]. This substantial genome expansion is most plausibly explained by TE accumulation, a pattern widely observed across other plant species [43]. Consistent with this interpretation, we detected striking differences in TE abundance and insertion patterns between wild *D. broteri* and the cultivated *D. caryophyllus* var. ‘Aili’. In *D. broteri*, TE insertions are predominantly confined to intergenic regions, whereas the cultivated variety exhibited a higher frequency of insertions within or near genes (exons, introns, and 1 kb flanking regions), despite having a lower overall TE content. Such contrasting TE insertion patterns may reflect historical differences in effective population sizes and the efficiency of purifying selection, both of which are expected to be reduced during domestication. However, given the comparative nature of our analysis, these patterns should be interpreted as correlational rather as direct evidence of domestication-driven TE proliferation. One possible explanation is that *D. broteri* has experienced tighter regulatory control of transposition activity, whereas cultivated carnations have undergone demographic bottlenecks, artificial selection, and stress-associated genomic responses during domestication. These processes can relax constraints on TE activity, facilitating bursts of retrotransposition and increasing the likelihood of insertions within genic regions [44]. TEs are well known to play a key role in generating genetic variation that can facilitate rapid phenotypic change and local adaptation [45], and they have been linked to domestication-related traits in several crops, including kernel pigmentation of maize [46] and fruit color and aroma in heirloom tomatoes [47]. In olive cultivars, domestication has similarly been associated with elevated retrotransposition activity and preferential insertions near genes [7]. In line with these observations, the recent peak of Gypsy and Copia LTR insertions in *D. caryophyllus* supports the occurrence of a retrotransposition burst that may be associated with domestication. By contrast, the extensive TE accumulation observed in wild *D. broteri* may reflect long-term exposure to environmental stress coupled with stronger purifying selection, promoting tighter control of retrotransposition and the selective removal of deleterious genic insertions. Nevertheless, disentangling the relative contributions of domestication, demographic history, and lineage-specific TE regulation will require population-level genomic analyses. Such data will be essential to determine whether the observed TE insertion patterns represent a general consequence of domestication in *Dianthus* or reflect species-specific evolutionary trajectories.

Gene family expansion/contraction analysis revealed marked differences between wild diploid *D. broteri* and the cultivated *Dianthus* MRCA. Due to the lack of reliable molecular divergence estimates for cultivated lineages, we approximated the divergence between wild and cultivated *Dianthus* using the estimated time of domestication. Consequently, these results should be interpreted as reflecting recent evolutionary dynamics associated with domestication and breeding history rather than true species divergence. In cultivated *Dianthus*, expanded families were enriched in growth-related functions, while contracted families were associated with secondary metabolism (such as scent production) and stress responses. In contrast, *D. broteri* showed expansion in gene families linked to secondary metabolism, aromatic compound catabolism, and stress response. These patterns align with artificial selection in *D. caryophyllus* for rapid growth, early flowering [13], and floral diversity [14]. While cultivated carnations lack the scent diversity of their wild relatives [33], *D. broteri* displays a floral scent dominated by terpenoids such as (E)-β-caryophyllene, (E)-nerolidol, and (E)-β-ocimene. Unlike in other genera such as *Rhododendron* [48], this enriched scent profile was not associated with an expansion of TPS genes, which were present in similar numbers in *D. broteri* and *D. caryophyllus* var. ‘Aili’. We hypothesized that uncontrolled TE activity in *D. caryophyllus* may have disrupted terpenoid biosynthesis. However, our analyses revealed no differences in TE insertions within MEP, MVA, or TPS genes. While this suggests that direct disruption is unlikely, the influence of the broader TE landscape on the epigenetic regulation of terpenoid gene expression cannot be ruled out [49]. Resource allocation away from scent biosynthesis may also contribute to the reduced aroma of cultivated carnations. Bright-colored flowers often exhibit diminished scent compared to pale ones, reflecting a trade-off between metabolic investment into color and aroma [48]. In *D. caryophyllus*, domestication-driven color variation is largely due to flavonoids (anthocyanins, chalcones, flavones, flavonols), carotenoids, and chlorophylls [50–52]. The MVA and MEP pathways generate the isoprenoid precursors isopentenyl diphosphate (IPP) and dimethylallyl diphosphate (DMAPP), which can be redirected toward carotenoid and chlorophyll biosynthesis in *D. caryophyllus* at the expense of volatile terpenoid production. Furthermore, flavonoid biosynthesis has been shown to influence terpenoid production in glandular trichomes of *S. lycopersicum* [53].

Additional evidence for the genomic regulation of floral scent in carnations comes from the pronounced differences in volatile emission between the eastern and western lineages of *D. broteri*. Comparative gene expression analyses of the terpenoid biosynthesis pathway revealed an overall upregulation of key genes in western populations, which exhibited higher levels of scent emission. Interestingly, several TPS genes showed constitutive expression across both lineages (Fig. S3), and some were even more highly expressed in eastern populations, despite their lower volatile output. This apparent decoupling between the levels of TPS transcript and the emission of volatiles suggests that transcriptional regulation alone cannot explain scent divergence. Our analysis of genetic variation in *DbrTPS18*, the most highly and constitutively expressed (Figs 6, S3, and  S4), provides an additional explanatory framework. We observed lineage-specific nonsynonymous changes, including those near conserved catalytic motifs and within relevant functional and structural domains of the protein. Such substitutions may affect enzyme conformation, substrate affinity, or catalytic efficiency, thereby modulating terpene output independently of transcript levels [54–56]. Substitutions were also identified within the predicted plastid transit peptide, and given that monoterpene synthases depend on plastid localization, variation in this region could influence targeting efficiency and, consequently, terpene production [57]. Altogether, molecular differences in terpenoid biosynthesis between *D. broteri* lineages may reflect divergent ecological and evolutionary pressures acting on floral scent across the distinct habitats occupied by eastern and western lineages. Variation in climatic conditions, pollinator assemblages, or antagonistic interactions may impose contrasting selective regimes on volatile production and emission, promoting lineage-specific adaptive trajectories [18, 19]. In this context, selection acting on enzyme functionality may facilitate rapid shifts in scent phenotype. While experimental validation is necessary to directly test the effects of the changes on the enzyme, the data are consistent with the notion that structural variation at the protein level, rather than differences in transcription levels, may contribute to the reduced terpene emissions in the eastern lineage.

At the same time, alternative or complementary mechanisms cannot be excluded. Terpenoids may be synthesized but retained in floral tissues rather than emitted. In *A. thaliana*, for example, the cytochrome P450 enzyme CYP706A3 constitutively oxidizes terpenes and sesquiterpenes into terpene oxides that accumulate in floral buds and function as insect repellents [58]. Previous research has reported that endogenous terpenoids are prevalent compounds in certain *Dianthus* species [17]. In the same way, the East *D. broteri* plants could be producing terpenoids that are being transported and retained in other flower tissues like in *Petunia thaliana,* where a tube-specific TPS produces sesquiterpenes that accumulate in the stigma for defense purposes [59]. Some TPS characterized in *D. broteri* are tissue-specific, like *TPS05*, *TPS08*, and *TPS34*, which synthesize the defense sesquiterpene α-isocomene exclusively in roots [60,61]. Contrary to TPS genes, all differentially expressed genes of the MVA or MEP pathway genes were overexpressed in the West, indicating that upstream precursor availability could be an additional limiting factor in eastern *D. broteri* lineage scent production.

In conclusion, we present a high-quality pseudo-chromosome level reference genome of the wild carnation *D. broteri*, providing new insights about how domestication shapes genome structure by activation of transposable elements. Furthermore, we investigated the volatilome of *D. broteri* and characterized candidate genes responsible for terpenoid production, focusing on the differences in expression between scented and unscented lineages of this wild carnation species. These findings support that regulation of floral scent in *Dianthus* could also occur at the first steps of the terpenoid biosynthetic pathway and/or through loss of functionality of specific terpene synthases. Moreover, they reinforce the idea that the loss of scent in *D. caryophyllus* may result from metabolic reallocation toward pigmentation, as increased flux toward flavonoids, carotenoids, and chlorophylls during domestication could reduce substrate availability for volatile terpenoid synthesis. Results are useful for future applications on domesticated carnation as a source of candidate genes and biosynthetic routes to improve *D. caryophyllus* breeding [17]. These applications highlight the importance of having genomic information and resources of the wild relatives of domesticated species.

## Material and methods

### Plant material and sequencing

*D. broteri* reference genome was constructed from an autogamous individual, originating from São Brás de Alportel (Southern Portugal). All DNA and RNA samples were taken from individuals kept in a climate chamber for optimal growth and bloom (16 h light 25°C/8 h dark 21°C regimen with 50% humidity) at the greenhouse facilities of the research center ‘Centro de Investigación, Tecnología e Innovación de la Universidad de Sevilla’ (CITIUS II), and then stored at −80°C until extraction. For genome assembly, high-quality DNA was extracted from fresh leaves following a modified version of an existing protocol [62] (dx.doi.org/10.17504/protocols.io.j8nlky116g5r/v1). Non-size selected SMRTbell libraries of extracted DNA were prepared and sequenced with PacBio Sequel II platform. For Illumina short read sequencing, used for polishing and scaffolding, genomic DNA was extracted using Qiagen DNeasy Plant Kit. Libraries were prepared using TruSeq DNA PCR-Free Kit with an insert size of 550 bp and sequenced using Illumina Novaseq 6000 technology. RNA was isolated from petal, leaf, and root samples using the Direct-zol RNA Miniprep kit (ZymoResearch) following manufacturer recommendations. Subsequently, RNA sequencing libraries were constructed using the TruSeq Stranded mRNA LT Sample Prep Kit. Libraries were sequenced on Illumina Novaseq 6000 platform, as 150 bp paired end. All DNA and RNA quality were verified using Qubit fluorometric quantification and Nanodrop spectrophotometer.

### Genome assembly

Nuclear DNA content of *D. broteri* was estimated by FCM using 100 mg of fresh leaf material taken from individuals stored in the greenhouse. *Pisum sativum* ‘Ctirad’ (9.09 pg/2C) was used as internal standard. FCM measurements were performed following the protocol previously described [29]. Additionally, genome size was estimated using Jellyfish K-mer analysis (*K* = 21), obtaining heterozygosity and repeat content of the studied genome. Results were visualized using Genomescope 2.0 [63].

PacBio sequences were initially assembled using Canu v2.0 [64]. Draft assembly heterozygosity was removed by running two rounds of Purge_Haplotigs v1.1.1 [65], using the ignore repeats (*−r*) option on the second round of purging to avoid collapsing of repeat regions and excessive purging due to high genomic repetitiveness. Further purging did not improve the assembly, rather caused important reduction of genome size accompanied by a decrease in completeness (Table S8). Obtained draft genome was scaffolded using the same long reads with LRScaf v1.1.12 [66] and then polished using short reads with POLCA v4.0.9, part of MaSuRCA pipeline [67], with a total of three scaffolding–polishing rounds (Table S9). A final step of homology-based scaffolding was performed using Ragtag [68], with *D. caryophylus* ‘Aili’ as reference [9].

Genome assembly statistics were retrieved using QUAST [69]. QV was obtained with Merqury v1.3 [30], analyzing the K-mer counts (*K* = 21) of Illumina pair-end gDNA reads. Genome assembly completeness was tested using BUSCO v5.0.0 [70] with eudicotyledons_odb10 database. Quality and continuity of the assembly was tested aligning DNA Illumina using BWA [71], and mRNA reads aligned using STAR [72]. We also evaluated the assembly continuity by calculating the LAI score using LTR_retriever [41].

### Repeat annotation and analysis

We performed transposable elements and repeat annotation to mask them for gene annotation. *De novo* repeat libraries were constructed using RepeatModeler II v2.0.3 (–LTRStruct option; [73]). These were separated into known and unknown families, and used together with RepeatMasker edition of RepBase version 20 181 026 to identify repetitive elements in the *D. broteri* genome using Repeat Masker v4.1.2-p1 [74]. The search engine used was NCBI (−e ncbi). We masked simple repeats first (−noint -xsmall) and then left apart when masking complex repeats (−nolow option).

Predictions of LTR-RTs within *Dianthus* species were calculated using LTR harvest [75], available through GenomeTools v1.2.1. We applied the same repeat annotation and LTR detection pipeline to all four studied genomes (RepeatMasker–LTRharvest–LTR_retriever) to ensure methodological consistency and comparability across assemblies. The fragmented assemblies of *D. caryophyllus* ‘Francesco’ and *D. sylvestris* impeded the identification of intact LTR elements in these species. Consequently, this analysis was conducted on *D. caryophyllus* ‘Aili’ and *D. broteri* genomes, representing cultivated and wild *Dianthus*, respectively. Intact LTR-RTs were classified using LTR retriever v2.9., which also calculates insertion of the repetitive elements.

### Structural and functional gene annotation

Gene models were predicted using both BRAKER v2.1.6 [76, 77] and MAKER 2 v3.01.03 [78]. First, we mapped RNA-seq data to the masked genome using STAR to obtain splicing information with *bamtohints* script from AUGUSTUS v3.5.0 [79, 80]. Splice information together with single-copy proteins produced by BUSCO (−long option) were fed into BRAKER as evidence, obtaining gene models and training AUGUSTUS at the same time.

Final gene predictions were obtained after three rounds of MAKER 2. For the first annotation round we used (i) the *D. broteri* masked transcriptome assembled with Trinity v2.14.0 [81] from five RNA-seq libraries, Transdecoder v5.7.1 (https://github.com/TransDecoder/TransDecoder.git) predicted coding sequences (CDS) from transcriptome and other *Dianthus* CDS from NCBI as EST evidence; (ii) *D. broteri* proteins from NCBI, available *Dianthus* protein sequence from RefSeq-NCBI and eudicotyledons protein information from Uniprot, together with Transdecoder peptides and BUSCO predicted proteins as protein homology evidence; (iii) repeat annotation from RepeatMasker, and (iv) gene models from BRAKER. Obtained gene models were used to train SNAP version 2006-07-28 [82], which were used together with AUGUSTUS trained gene models from BRAKER in the second annotation round. A final round of annotation was run iteratively, training gene models with SNAP again with round two output. BRAKER and MAKER gene models were evaluated with BUSCO. The final gene annotation was obtained after merging unique gene models with AGAT (https://github.com/NBISweden/AGAT.git).

Functional annotation of the final predicted gene set was performed with OmicsBox 3.0.30. We used DIAMOND blastx [37] against the Magnoliopsida database stand 2023-02-01, and GOs version 2022.08 for GO mapping [83]. The rest of the parameters were set to default. Finally, a Cloud InterProScan version 5.63–95.0 [84] was performed, and the InterProScan GOs were validated and merged with the Blast annotations.

### Phylogenetic analysis

Protein sequences of nine species along with cultivated and wild carnations were used to build the species tree (Table S10). Orthofinder v2.5.5 was used to obtain orthogroups and estimate the gene trees [85]. Orthologs were identified using DIAMOND [37] and clustered into orthogroups and paralagous groups, and then single-copy genes were selected. Multiple sequence alignment (MSA) was performed using MAFFT and the rooted species phylogenetic tree was inferred by FastTree maximum likelihood methods as part of the Orthofinder pipeline.

### Gene family evolution and gene ontology enrichment analysis

Once the species phylogenetic tree was ready, we calibrated the divergence times of nodes using secondary calibration points obtained from TimeTree [86] (Table S3), which report divergence time estimates derived from published molecular and fossil-calibrated studies. As there are no reported estimates for the divergence time between *D. caryophyllus* and wild *Dianthus* species, we considered *D. sylvestris* to be the closest wild relative and approximated their divergence time based on the estimated timing of carnation domestication (~2000 years ago; [9]). Minimum and maximum age constraints were set as hard bounds for all calibrated nodes, except for the root node (node 14, Fig. 2). We then used 145 single-copy OGs to reconstruct a species tree and the ‘ape’ R package v5.8 [87] to make it ultrametric. This was used as an input into CAFE v5.1.1 [88] to estimate gene family expansion, with three gamma categories for the birth–death model (−k 3) and a significance level of 0.05 (default), using a base error model generated from test runs (−e option).

To infer expanded and contracted functions associated with diversification between cultivated and wild *Dianthus* species, we performed a GO enrichment analysis of the expanded and contracted gene families of *D. broteri* and *D. caryophyllus* MRCA, including gene families of both ‘Aili’ and ‘Francesco’ varieties of *D. caryophyllus* included in this research. The GO annotations for both species, generated by OmicsBox, were assigned into the gene families and used as an input for ‘ClusterProfiler’ R package v4.12 [89], which tested for overrepresented GO terms. Full ontologies were built using the function ‘buildGOmap’ and the GO enrichment analysis was performed using the ‘enricher’ with FDR correction and 0.05 as *P*-value cut-off. The results were uploaded to REVIGO [90] to select the biological process (BP) GO terms and summarize the output for easier visualization.

### Volatilome characterization by GC–MS analysis

Floral volatiles of *D. broteri* were collected from 20 individuals by enclosing a flower within a polyester bag and capturing emitted volatiles with an adsorbent tube containing 1.5 mg of Tenax-TA and 1.5 mg of Carbotrap B (both Supelco, Bellefonte, PA, USA) [27]. Collection process was facilitated by a rotary vane pump (G12/01 EB; Rietschle Thomas Inc., Puchheim, Germany; 200 ml/min flow rate) for 15 min. To minimize biological variation, floral scent sampling was standardized by both floral developmental stage and time of day. *D. broteri* exhibits protandrous dichogamy, with flowers transitioning from a male to a female phase. Floral scent was collected at the end of the male phase, just before stigma exposure (~5 days after anthesis). To capture the whole volatilome*,* samples were taken during standardized daytime (10:00 a.m.–12:00 p.m.) and nighttime (8:00–10:00 p.m.) windows, with 10 samples per lineage (five days and five nights) [24]. After volatile collection, petals from sampled flowers were harvested and immediately frozen for RNA extraction and sequencing as described previously (Table S4). Samples from leaves and ambient air were used as negative controls for VOC quantification.

Collected scent samples were analyzed via thermal desorption-gas chromatography–mass spectrometry (GC–MS) and the acquired data were analyzed using GCMS solution. Obtained compounds were tentatively identified by comparison of linear retention indices (RI, based on a series of commercially available n-alkanes C7–C20; van Den Dool and Dec. Kratz [91]) and a match of mass spectra to spectra available in the reference libraries ADAMS, ESSENTIALOILS-23P, FFNSC 2, and W9N11. Whenever possible, compound identification was further supported using a database generated from synthetic standards available in the Plant Ecology Laboratory of the University of Salzburg. Peak areas (total ion current) of the single compounds were used to calculate the total amount of scent per sample and the relative amounts of single compounds. Volatile compound classification was conducted using the R package Chemodiv v0.2.0 package [92]. Relationships among VOCs were elucidated by hierarchical clustering and a principal component analysis (PCA). Furthermore, with a Permutational Multivariate Analysis of Variance Using Distance Matrix (PERMANOVA) on relative abundances of compounds, we explored variations in scents attributed to geographic location (East vs West), using the vegan v2.6–4 package [93]. In subsequent analyses, we focused on terpenoid pathway compounds and conducted Kruskal–Wallis tests to assess differences in emissions of major terpenoid groups between the East and West lineages.

### Terpenoid biosynthetic pathway and terpene synthases identification and localization

We used the KIPEs v3.2.4 [35] to identify structural genes involved in terpenoid biosynthesis from *D. broteri* protein sequences. A custom Python script was applied to identify candidate orthologous genes linked to terpenoid biosynthesis, with *A. thaliana* genome from ENSEMBL serving as the sources for baits. Conserved amino acid residues were determined using MAFFT v7.490 alignments [94].

TPS genes were identified with HMMER v3.4 [36] using two specific TPS domains (PF03936 and PF01397) from Pfam [95]. *Arabidopsis thaliana* TPS (AtTPS) protein sequences, retrieved from TAIR10.1 genome (GCF_000001735.4, NCBI), were aligned with *D. broteri* proteins using DIAMOND. Resulting *D. broteri* TPS (DbrTPS) physical properties were obtained using ExPASy [96]. DeepLoc 2.0 (services.healthtech.dtu.dk) and plant-mpLoc (www.csbio.sjtu.edu.cn) were used to determine the cellular locations of proteins. Additionally, CLEAN was used as validation tool, performing enzyme functional annotation based on machine learning techniques [40].

To classify obtained TPS genes from both *D. caryophyllus* and *D*. *broteri* we constructed a phylogenetic tree with TPS protein sequences from *A. thaliana* from Phytozome. Cobalt [97] was used for alignment based on conserved motifs, and ModelFinder [98] identified the best nucleotide substitution model. IQ-TREE 2 v2.3.4 [38] was then utilized to infer maximum likelihood (ML) trees with this model with 1000 ultrafast bootstrap replicates (−bb 1000). AthTPS genes annotation were extrapolated to the DbrTPS and DcaTPS for classification [99]. MEME Suite was applied with default parameters to identify conserved motifs within the TPS sequences [39].

### Differential expression analysis

To examine differential expression among tissues, we analyzed RNAseq from three samples of each tissue (root, leaf and petals). For differential expression of scent biosynthesis genes among East and West lineages, we compared RNAseq from same petals used for scent collection, with 10 samples for each lineage. Fastp v0.23.3 [100] was used for adapter removal and quality trimming. Any possible rRNA contamination was detected and filtered out using SortMeRNA v4.3.2 [101]. Quality of the trimmed reads was checked using FastQC v0.11.9 [102]. Subsequently, the sequence reads were aligned to the *D. broteri* reference genome using the STAR v2.7.10b with the ‘–quantMode’ argument to obtain the number of reads per gene [72] Differential expression (DE) analysis was conducted with R package edgeR v4.2.2 [103]. Two separate DE analyses were performed using different datasets. First, we analyzed differential tissue-specific expression. Second, differential expression of samples from East and West lineages was performed using flower tissue samples only. The threshold criteria for identifying DE genes were set at an FDR *P*-value of .05 and a log fold change of 1.5. Further visualization of the results was achieved via heatmap using *z*-scores, to standardize the data, being able to compare across the different groups.

### Variant calling and association analyses of terpenoid biosynthesis genes

Variant discovery from RNA-seq data was focused on genes involved in the terpenoid biosynthetic pathway. SNPs were called following GATK v4 best practices [104] from previous STAR alignments. Raw variant calls were restricted to biallelic SNPs and filtered (QD < 2.0, FS > 60.0, SOR > 3.0, MQ < 40.0, MQRankSum < −12.5, and ReadPosRankSum < −8.0) using bcftools [105]. Genotype-level filters were then applied by masking low-confidence genotypes (DP < 5 or GQ < 20) to missing, and variants with >20% missing genotypes (F_MISSING >0.2) were excluded. To reduce the influence of rare alleles, a minor allele count filter was applied (MAC ≥ 4). Variant annotation with SnpEff [106] revealed that a single gene (*DIABRO_021246*) within this pathway accounted for >50% of all detected polymorphisms and subsequent analyses were therefore restricted to this locus. Lineage-specific SNPs, defined as private variants present in ≥80% of individuals within a lineage, were identified by contrasting allele presence among genetic lineages. The functional impact of nonsynonymous variants was also assessed using SIFT [107], and potential effects on chloroplast transit peptides were evaluated with ChloroP [108]. Genetic structure was summarized using PCA and a genetic relatedness matrix inferred from LD-pruned SNPs with SNPRelate [109]. Associations between candidate SNPs and the main terpenoid compounds, (E)-β-Caryophyllene, (E)-β-Ocimene, and (E)-Nerolidol, were tested using single-variant score tests implemented in GENESIS [110], with FDR correction applied for multiple testing (FDR < 0.05). The amino acid sequence of the candidate terpene synthase (*DbrTPS18*) was analyzed to identify conserved domains and catalytic motifs associated with plant TPS enzymes, with emphasis on the TPS-g subfamily. Conserved catalytic motifs characteristic of class I TPS enzymes, including the aspartate-rich DDXXD motif and the NSE/DTE motif, were identified by manual inspection and motif searches using ScanProsite.

## Supplementary Material

Web_Material_uhag130

## Data Availability

Genomic data underlying this study are available in NCBI GenBank BioProject PRJNA1024664. The code of the genomic and statistical analyses carried out are available in the GitHub repository jesuspi/DbroteriGenome at https://doi.org/10.5281/zenodo.19629020.
