# Supplementary material for: Genomic basis of scent loss and functional divergence in wild and cultivated carnations (Dianthus spp.)
Source: Hortic Res. 2026 Apr 7;13(8):uhag130. doi: 10.1093/hr/uhag130 (PMC13412721; doi:10.1093/hr/uhag130)
Supplement: Web_Material_uhag130 [file web_material_uhag130.zip › SUP_TABLES.pdf]

Supplementary table 1: Abundance of repetitive elements in wild (*Dianthus broteri* and *D. sylvestris*) and cultivated (*D. caryophyllus* var. 'Aili' and 'Francesco') carnations based on Repeat Masker repeat annotation.

			Dianthus broteri			***Dianthus caryophyllus* Aili**			***Dianthus caryophyllus* Francesco**			***Dianthus sylvetris***		
**Class**	**Order**	**Superfamily**	**count**	**total_length**	**percentage**	**count**	**total_length**	**percentage**	**count**	**total_length**	**percentage**	**count**	**total_length**	**percentage**
**Class I/Retroelen**	DIRS	Ngaro	NA	NA	NA	NA	NA	NA	NA	NA	NA	534	216386	0.04
	LINE	CRE	11644	3140924	0.36	1701	897297	0.15	1909	917299	0.16	1884	569486	0.10
		L1	83223	22004311	2.51	22987	19572307	3.36	26917	15742178	2.76	21168	8475315	1.46
		R1	NA	NA	NA	3386	7908932	1.36	2944	3065316	0.54	71	10132	0.00
		RTE	7062	1694642	0.19	989	119965	0.02	1334	161689	0.03	6278	700481	0.12
	LTR	Caulimovirus	5887	1837074	0.21	15196	2825933	0.49	16806	2679210	0.47	7704	1773122	0.30
		Copia	157877	82431915	9.41	130511	58273320	10.01	119073	38805652	6.80	88723	25631851	4.40
		Gypsy	600100	444823943	50.77	333093	228556620	39.24	272115	125456346	22.00	133984	50266422	8.63
		ERV	NA	NA	NA	769	192467	0.03	942	180926	0.03	5569	1129542	0.19
		Pao	22	1500	0.00	NA	NA	NA	NA	NA	NA	46	31172	0.01
		TRIMs	4883	839706	0.10	4102	643734	0.11	3591	483978	0.08	4671	792898	0.14
		Unclassified	4449	622952	0.07	9432	1464761	0.25	12209	1795421	0.31	11435	1569337	0.27
	SINE	NA	4525	812066	0.09	22335	5649067	0.97	20988	4443621	0.78	3667	502564	0.09
**Class II/DNA**	Helitron	Helitron/RC	45694	9473712	1.08	8406	2252865	0.39	5770	1724624	0.30	39749	6815240	1.17
	TIR-DDE/E	EnSpm/CACTA	35319	11068776	1.26	32061	9043359	1.55	26643	6241739	1.09	23415	4061629	0.70
		Kolobok-T2	881	274001	0.03	NA	NA	NA	NA	NA	NA	NA	NA	NA
		MULE-MuDR	71389	22264325	2.54	85290	16813340	2.89	90011	15812764	2.77	29580	7795706	1.34
		PIF-Harbinger	9171	1489417	0.17	12992	2958992	0.51	11818	3004720	0.53	8504	1629076	0.28
		PiggyBac	NA	NA	NA	NA	NA	NA	NA	NA	NA	615	59839	0.01
		Tc-Mariner	2817	622970	0.07	24349	4317075	0.74	30692	5133356	0.90	30642	5138301	0.88
		Unclassified	1264	202351	0.02	1383	160836	0.03	1684	203075	0.04	5387	651675	0.11
		Zisupton	1825	336767	0.04	2336	610438	0.10	3055	743203	0.13	1456	322015	0.06
		hAT	97258	19916376	2.27	69693	12780292	2.19	87321	15075702	2.64	75647	11792710	2.02
**Low_complexity**	Low_complexity	Low_complexity	105856	5936368	0.68	66661	3658530	0.63	59633	3180622	0.56	39560	2046846	0.35
**Satellite**	Satellite	Satellite	1619	363009	0.04	3131	476713	0.08	456	46417	0.01	348	35685	0.01
**Simple_repeat**	Simple_repeat	Simple_repeat	541896	29732961	3.39	328834	17230133	2.96	299100	14474871	2.54	205237	9861775	1.69
**Small RNA**	Small RNA	rRNA	760	549245	0.06	5124	6165631	1.06	361	64244	0.01	103	22845	0.00
		snRNA	128	48227	0.01	32	5397	0.00	36	5992	0.00	281	49267	0.01
		tRNA	1142	163549	0.02	620	44930	0.01	747	54137	0.01	60582	8807988	1.51
**Unknown**	Unknown	Unknown	218791	59848794	6.83	163872	37530208	6.44	183512	38718268	6.79	171428	36268406	6.23

Supplementary table 2: Insertion time of Long Terminal Repeat (LTR) subfamilies in *Dianthus broteri* and *D. caryophyllus* var. 'Aili'.

**LTR Clade**	**Subfamily**	***D. broteri***	***D. caryophyllus* 'Aili'**
**Gypsy**	Athila	146950	90890
	CRM	134013	43789
	Galadriel	74634	0
	Ogre	118150	43358
	Reina	155505	242153
	Retand	115212	40218
	Tekay	24310	24975
**Copia**	Ale	96830	0
	Alesia	930602	166983
	Angela	81252	76187
	Bianca	210941	177789
	Ikeros	83100	71672
	Ivana	94191	109162
	SIRE	87297	60190
	TAR	75431	114697
	Tork	75513	100334

Supplementary table 3: Calibration points of Orthofinder species phylogenetic nodes. Divergence times, derived from published molecular and fossil-calibrated studies, were obtained from TimeTree database

**Node**	**Divergence time (Mya)**	
	**Min**	**Max**
14	142.1	163.5
15	110.6	120
16	111.4	123.9
17	28	41.3
18	102	112.5
19	54.5	72
20	20.3	46.7
22	1.9	7
23	0.001	0.01
25	24.5	73.8

Suplemmentary table 4: Population information of *Dianthus broteri* individuals used in floral scent profile characterization and RNAseq differential expression analysis. Seeds from wild populations were collected and cultivated under controlled conditions at the at the greenhouse facilities of the research center "Centro de Investigación, Tecnología e Innovación de la Universidad de Sevilla" (CITIUS II). Scents were collected from the flowers which petals were then frozen for RNA extraction and sequencing. Herbarium accesion number refers to herbarium records of the University of Seville.

Sample ID	Lineage	Collection ToD	Population	Altitude (m)	Coordinates	Herbarium acc.
D026	East	Night	Zafarraya, Spain	1017	36º59'17"N/04º11'02"W	SEV 277753
D090	East	Night	Venta de Zafarraya, Spain	886	36º56'09"N/04º07'33"W	SEV 277754
D101	East	Day	Zafarraya, Spain	1017	36º59'17"N/04º11'02"W	SEV 277753
D106	West	Day	São Brás de Alportel, Portugal	368	37º09'14"N/07º50'02"W	SEV 216219
D112	West	Night	São Brás de Alportel, Portugal	368	37º09'14"N/07º50'02"W	SEV 216219
D114	East	Day	Laroles, Spain	1051	37º00'32"N/01º25'00"W	SEV 277752
D116	West	Day	São Brás de Alportel, Portugal	368	37º09'14"N/07º50'02"W	SEV 216219
D121	East	Night	Laroles, Spain	1051	37º00'32"N/01º25'00"W	SEV 277752
D125	East	Night	Mecina de Alfahar, Spain	819	36º59'56"N/03º04'09"W	SEV 277751
D127	East	Day	Venta de Zafarraya, Spain	886	36º56'09"N/04º07'33"W	SEV 277754
D144	East	Night	Mecina de Alfahar, Spain	819	36º59'56"N/03º04'09"W	SEV 277751
D152	East	Day	Mecina de Alfahar, Spain	819	36º59'56"N/03º04'09"W	SEV 277751
D158	East	Day	Mecina de Alfahar, Spain	819	36º59'56"N/03º04'09"W	SEV 277751
D167	West	Night	Archidona, Spain	748	37º06'58"N/04º18'53"W	SEV 277773
D172	West	Night	São Brás de Alportel, Portugal	368	37º09'14"N/07º50'02"W	SEV 216219
D177	West	Day	Archidona, Spain	748	37º06'58"N/04º18'53"W	SEV 277773
D185	West	Night	São Brás de Alportel, Portugal	368	37º09'14"N/07º50'02"W	SEV 216219
D186	West	Day	São Brás de Alportel, Portugal	368	37º09'14"N/07º50'02"W	SEV 216219
D206	West	Day	São Brás de Alportel, Portugal	368	37º09'14"N/07º50'02"W	SEV 216219
D220	West	Night	São Brás de Alportel, Portugal	368	37º09'14"N/07º50'02"W	SEV 216219

Supplementary table 5: Mean relative amount (%) of floral scent compounds of the West and East lineages of *Dianthus broteri*. RI: linear retention index. Compound classification based on Knudsen et al 2006.

Compound	RI	Pathway	East	West	Total
(*E*)-2-Hexenal*	851	Fatty acid derivati	0.000	<0,001	<0,001
(*Z*)-3-Hexen-1-ol*	854	Fatty acid derivati	0.052	0.009	0.010
1-Hexanol*	865	Fatty acid derivati	0.000	<0,001	<0,001
2-Heptanone*	889	Fatty acid derivati	0.003	0.000	<0,001
unk_962	962	Unknowns	0.000	0.002	0.002
Verbenene	971	Monoterpenoids	0.000	<0,001	<0,001
β-Myrcene*	992	Monoterpenoids	0.000	0.015	0.015
(*Z*)-3-Hexenyl acetate*	1005	Fatty acid derivati	0.168	0.011	0.018
unk_1010	1010	Unknowns	0.031	0.016	0.016
Benzyl alcohol*	1036	Benzenoids	0.000	0.003	0.003
(*Z*)-β-Ocimene*	1039	Monoterpenoids	0.011	0.004	0.004
Lavender lactone*	1044	Miscellaneous cyc	0.000	<0,001	<0,001
(*E*)-β-Ocimene*	1050	Monoterpenoids	0.199	0.086	0.090
unk_1056	1056	Unknowns	0.000	0.001	0.001
unk_1082	1082	Unknowns	0.000	0.002	0.001
2-Nonanone*	1092	Fatty acid derivati	0.013	0.000	<0,001
p-Cymenene	1095	Monoterpenoids	0.009	0.004	0.004
Rosefuran	1098	Monoterpenoids	0.000	<0,001	<0,001
unk_1111	1111	Unknowns	0.004	0.003	0.003
allo-Ocimene*	1132	Fatty acid derivati	0.004	0.002	0.002
(*E*)-Ocimene epoxide*	1143	Monoterpenoids	0.000	<0,001	<0,001
neo-allo-Ocimene*	1145	Monoterpenoids	0.002	<0,001	<0,001
Benzyl acetate*	1169	Benzenoids	0.000	0.006	0.006
p-Mentha-1,5-dien-8-ol	1175	Monoterpenoids	0.003	0.002	0.002
(*Z*)-3-Hexenyl butyrate*	1186	Fatty acid derivati	0.000	<0,001	<0,001
unk_1217	1217	Unknowns	0.000	0.001	0.001
unk_1225	1225	Unknowns	0.000	<0,001	<0,001
(*Z*)-3-hexenyl 2-methylbutanoate*	1233	C5-branched chai	0.000	<0,001	<0,001
2-Undecanone*	1295	Fatty acid derivati	0.032	0.000	0.001
unk_1428	1428	Unknowns	0.000	0.005	0.005
Isocaryophyllene	1430	Sesquiterpenoids	0.000	0.002	0.001
unk_1434	1434	Unknowns	0.000	<0,001	<0,001
(*E*)-β-Caryophyllene*	1445	Sesquiterpenoids	0.000	0.404	0.387
(*E*)-β-Farnesene*	1461	Sesquiterpenoids	0.000	0.012	0.012
unk_1466	1466	Unknowns	0.000	0.002	0.002
α-Caryophyllene*	1479	Sesquiterpenoids	0.000	0.015	0.014
2-Tridecanone*	1497	Fatty acid derivati	0.261	0.000	0.011
(*Z*,*Z*)-α-Farnesene*	1498	Sesquiterpenoids	0.000	<0,001	<0,001
2-Tridecanol*	1503	Fatty acid derivati	0.010	0.000	<0,001
(*E*,*E*)-α-Farnesene*	1513	Sesquiterpenoids	0.000	0.004	0.004
β-Bisabolene*	1521	Sesquiterpenoids	0.000	0.001	0.001
unk_1561	1561	Unknowns	0.000	<0,001	<0,001
(*E*)-Nerolidol*	1571	Sesquiterpenoids	0.000	0.301	0.288
Caryophyllene oxide_derivative 1	1580	Sesquiterpenoids	0.000	<0,001	<0,001
(*Z*)-3-Hexenyl benzoate	1585	Benzenoids	0.000	0.002	0.002
unk_1601	1601	Unknowns	0.000	0.003	0.003
Caryophyllene oxide derivative 2*	1606	Sesquiterpenoids	0.000	<0,001	<0,001
unk_1615	1615	Unknowns	0.000	0.005	0.005
unk_1666	1666	Unknowns	0.000	<0,001	<0,001
unk_1679	1679	Unknowns	0.000	0.002	0.002
unk_1684	1684	Unknowns	0.000	0.001	0.001
unk_1696	1696	Unknowns	0.000	0.002	0.002
2-Pentadecanone*	1701	Fatty acid derivati	0.060	0.000	0.003

unk_1705	1705	Unknowns	0.002	<0,001	<0,001
(*E*,*E*)-Farnesol*	1731	Sesquiterpenoids	0.031	<0,001	0.002
unk_1740	1740	Unknowns	0.000	0.002	0.002
Benzyl benzoate*	1787	Benzenoids	0.079	0.026	0.028
Benzyl salicylate	1896	Benzenoids	0.021	0.035	0.034
2-Heptadecanone*	1904	Fatty acid derivati	0.005	0.000	<0,001

*Identification of compounds marked with an asterisk is based on synthetic standards

Suplemmentary table 6: Population information of *Dianthus broteri* individuals used for differential expression analysis of terpenoids genes among tissues. Herbarium accession number refers to herbarium records of the University of Seville.

Sample ID	Tissue	Population	Altitude	Coordinates	Herbarium acc.
2RALB	Root	Mecina de Alfahar, Spain	819	36º59'56"N/03º04'09"W	SEV 277751
2RLAR	Root	Laroles, Spain	1051	37º00'32"N/01º25'00"W	SEV 277752
2RSBR	Root	São Brás de Alportel, Portugal	2	37º09'14"N/07º50'02"W	SEV 216219
2H0202	Leaf	Laroles, Spain	1051	37º00'32"N/01º25'00"W	SEV 277752
2H0203	Leaf	Laroles, Spain	1051	37º00'32"N/01º25'00"W	SEV 277752
2H0206	Leaf	São Brás de Alportel, Portugal	2	37º09'14"N/07º50'02"W	SEV 216219
D106	Petal	São Brás de Alportel, Portugal	2	37º09'14"N/07º50'02"W	SEV 216219
D108	Petal	São Brás de Alportel, Portugal	2	37º09'14"N/07º50'02"W	SEV 216219
D177	Petal	Archidona, Spain	748	37º06'58"N/04º18'53"W	SEV 277773

Supplementary table 7: Functional annotation of genetic variants predicted using SnpEff. The table summarizes the effects of detected SNPs on gene models, including genomic location, affected gene and transcript identifiers, variant type, predicted impact category (high, moderate, low, or modifier), and functional consequence according to SnpEff annotations. Impact classifications follow SnpEff criteria based on the predicted severity of the variant on protein structure and function. Additional fields report annotation details such as codon and amino acid changes, affected protein regions, and relevant functional features.

**Locus**	**Allele**	**Annotation**	**Putative_impact**	**Gene Name**	**Feature type**	**Transcript biotype**	**Rank / total**	**HGVS.c**	**HGVS.p**	**cDNA_pos/cDNA_len**	**CDS_posi/CDS_len:**	**Protein_pos/Protein_len**
diaBro_01:59494975	C	splice_region_variant&intron_variant	LOW	DIABRO_002789.1	transcript	protein_coding	1/1	c.137-8T>C	-	-	-	-
diaBro_01:59495378	G	missense_variant	MODERATE	DIABRO_002789.1	transcript	protein_coding	2/2	c.532A>G	p.Asn178Asp	532/1107	532/1107	178/368
diaBro_04:1068671	A	synonymous_variant	LOW	DIABRO_010246.1	transcript	protein_coding	15/15	c.6609C>T	p.Ala2203Ala	6609/6711	6609/6711	2203/2236
diaBro_04:1070164	T	synonymous_variant	LOW	DIABRO_010246.1	transcript	protein_coding	13/15	c.6036C>A	p.Gly2012Gly	6036/6711	6036/6711	2012/2236
diaBro_04:1076387	A	synonymous_variant	LOW	DIABRO_010246.1	transcript	protein_coding	10/15	c.4317C>T	p.Asp1439Asp	4317/6711	4317/6711	1439/2236
diaBro_04:44299038	A	synonymous_variant	LOW	DIABRO_012458.1	transcript	protein_coding	8/10	c.1617G>T	p.Pro539Pro	1905/2742	1617/2127	539/708
diaBro_04:44299098	C	synonymous_variant	LOW	DIABRO_012458.1	transcript	protein_coding	8/10	c.1557A>G	p.Pro519Pro	1845/2742	1557/2127	519/708
diaBro_04:44299143	A	synonymous_variant	LOW	DIABRO_012458.1	transcript	protein_coding	8/10	c.1512C>T	p.Asp504Asp	1800/2742	1512/2127	504/708
diaBro_04:44299361	G	synonymous_variant	LOW	DIABRO_012458.1	transcript	protein_coding	7/10	c.1389T>C	p.Phe463Phe	1677/2742	1389/2127	463/708
diaBro_04:44302368	C	synonymous_variant	LOW	DIABRO_012458.1	transcript	protein_coding	2/10	c.144A>G	p.Ser48Ser	432/2742	144/2127	48/708
diaBro_06:76586839	A	missense_variant	MODERATE	DIABRO_021246.1	transcript	protein_coding	6/6	c.1699C>T	p.Pro567Ser	2065/2088	1699/1722	567/573
diaBro_06:76587469	G	missense_variant	MODERATE	DIABRO_021246.1	transcript	protein_coding	5/6	c.1355T>C	p.Val452Ala	1721/2088	1355/1722	452/573
diaBro_06:76587685	A	missense_variant	MODERATE	DIABRO_021246.1	transcript	protein_coding	4/6	c.1234C>T	p.Leu412Phe	1600/2088	1234/1722	412/573
diaBro_06:76587712	T	missense_variant	MODERATE	DIABRO_021246.1	transcript	protein_coding	4/6	c.1207G>A	p.Asp403Asn	1573/2088	1207/1722	403/573
diaBro_06:76587736	T	missense_variant	MODERATE	DIABRO_021246.1	transcript	protein_coding	4/6	c.1183G>A	p.Asp395Asn	1549/2088	1183/1722	395/573
diaBro_06:76587762	C	missense_variant	MODERATE	DIABRO_021246.1	transcript	protein_coding	4/6	c.1157C>G	p.Ser386Cys	1523/2088	1157/1722	386/573
diaBro_06:76587768	T	missense_variant	MODERATE	DIABRO_021246.1	transcript	protein_coding	4/6	c.1151G>A	p.Arg384Lys	1517/2088	1151/1722	384/573
diaBro_06:76587789	A	missense_variant	MODERATE	DIABRO_021246.1	transcript	protein_coding	4/6	c.1130C>T	p.Thr377Ile	1496/2088	1130/1722	377/573
diaBro_06:76587802	G	missense_variant	MODERATE	DIABRO_021246.1	transcript	protein_coding	4/6	c.1117T>C	p.Tyr373His	1483/2088	1117/1722	373/573
diaBro_06:76587818	A	synonymous_variant	LOW	DIABRO_021246.1	transcript	protein_coding	4/6	c.1101C>T	p.Ala367Ala	1467/2088	1101/1722	367/573
diaBro_06:76587836	G	synonymous_variant	LOW	DIABRO_021246.1	transcript	protein_coding	4/6	c.1083T>C	p.Leu361Leu	1449/2088	1083/1722	361/573
diaBro_06:76587904	T	missense_variant	MODERATE	DIABRO_021246.1	transcript	protein_coding	4/6	c.1015G>A	p.Ala339Thr	1381/2088	1015/1722	339/573
diaBro_06:76588888	T	intron_variant	MODIFIER	DIABRO_021246.1	transcript	protein_coding	2/5	c.475+127G>A	-	-	-	-
diaBro_06:76588890	T	intron_variant	MODIFIER	DIABRO_021246.1	transcript	protein_coding	2/5	c.475+125C>A	-	-	-	-
diaBro_06:76589052	G	synonymous_variant	LOW	DIABRO_021246.1	transcript	protein_coding	2/6	c.438T>C	p.Phe146Phe	804/2088	438/1722	146/573
diaBro_06:76589072	G	missense_variant	MODERATE	DIABRO_021246.1	transcript	protein_coding	2/6	c.418T>C	p.Tyr140His	784/2088	418/1722	140/573
diaBro_06:76636226	G	downstream_gene_variant	MODIFIER	DIABRO_021256.1	transcript	protein_coding		c.*4347T>C	-	-	-	-
diaBro_10:9858381	T	3_prime_UTR_variant	MODIFIER	DIABRO_028451.1	transcript	protein_coding	12/12	c.*275T>A	-	-	-	-
diaBro_13:48003571	C	missense_variant	MODERATE	DIABRO_035748.1	transcript	protein_coding	4/15	c.274C>G	p.Leu92Val	274/2295	274/2295	92/764

Supplementary table 8: *Dianthus broteri* assembly statistics across genome assembly and heterozygosity reduction. Genome assembly was performed with Canu, using PacBio CLR long reads after size filtering of reads shorter than 5kb. BUSCO completeness was assessed using the eudicotyledons_odb10 database. Haploidy was estimated using HapPy.

**Haplotig processing**	**Assembly size**	**N50**	**N contigs**	**BUSCO**						**Haploidy**
				**C**	**S**	**D**	**F**	**M**	**n**	
raw	1098330147	10997495	731	93.50%	57.80%	35.70%	1.50%	5.00%	2326	0.555
purge_haplotigs	949907989	12978766	235	93.40%	71.20%	22.20%	1.60%	5.00%	2326	0.683
purge_dups	517355021	21311061	92	78.2%	73.4%	4.8%	1.5%	20.3%	2326	0.982
2x purge_haplotigs	730776695	16308396	75	90.80%	75.90%	14.90%	1.50%	7.70%	2326	0.861
purge_haplotigs+purge_haplotigs with repeat masking*	875071064	13776570	121	93.30%	76.20%	17.10%	1.50%	5.20%	2326	0.769
purge_haplotigs+purge_haplotigs with repeat masking + purge_haplotigs	872986267	13776570	106	93.30%	76.20%	17.10%	1.50%	5.20%	2326	0.769

* Final version for pollishing and scaffolding

Supplementary table 9: *Dianthus broteri* assembly statistics during scaffolding and polishing. The assembly was scaffolded using PacBio long-reads with LRScaf, and polished using Illumina short-reads with POLCA. A total of three rounds were performed. After manual removing of duplicated contigs during scaffolding, a fourth round of homology-based scaffolding was performed using Ragtag and *D. caryophyllus* 'Aili' as reference.

**Assembly status**	**Assembly size**	**N50**	**n contigs**	**BUSCO**					
				**C**	**S**	**D**	**F**	**M**	**n**
Haplotig purged assembly	875071064	13776570	121	93.30%	76.20%	17.10%	1.50%	5.20%	2326
Scaffolding and pollishing round 1	987042767	16308201	111	93.40%	76.20%	17.20%	1.50%	5.10%	2326
Scaffolding and pollishing round 2	987528917	18945833	103	93.30%	76.10%	17.20%	1.50%	5.20%	2326
Scaffolding and pollishing round 3	987573394	19801982	100	93.30%	76.10%	17.20%	1.50%	5.20%	2326
Manually curation	876187314	16838809	97	93.40%	76.20%	17.20%	1.50%	5.10%	2326
Homology-based superscaffolding	876175788	53915179	34	93.40%	76.80%	16.60%	1.50%	5.10%	2326

Supplementary table 10: Accession numbers and references of protein resources used in phylogenomic analyses.

**Species**	**Source**	**Reference/Accesion**	**DOI**
Amaranthus hybridus	Dicot PLAZA v5.0.0	Montgomery et al., 2020	https://doi.org/10.1093/gbe/evaa177
Arabidopsis thaliana	Dicot PLAZA v5.0.0	Cheng et al., 2017	https://doi.org/10.1111/tpj.13415
Beta vulgaris	Dicot PLAZA v5.0.0	McGrath et al., 2020	https://doi.org/10.1101/2020.09.15.298315
Dianthus caryophyllus 'Aili'	CNGDdb	#CNA0004649	https://doi.org/10.3389/fpls.2023.1230836
Dianthus caryophyllus 'Francesco'	Carnation DB	http://carnation.kazusa.or.jp	https://doi.org/10.1093/dnares/dst053
Dianthus sylvestris	DRYAD	Fior, 2023	https://doi.org/10.5061/dryad.x0k6djhng
Gypsophila paniculata	CNGDdb	#CNA0050895	https://doi.org/10.1093/hr/uhac176
Heliosperma pusillum	NCBI	PRJNA739571	https://doi.org/10.1111/mec.16393
Oryza sativa	Dicot PLAZA v5.0.0	International Rice Genome Sequencing Project, 2005	https://doi.org/10.1038/nature03895
Petunia axillaris	Dicot PLAZA v5.0.0	Bombarely et al., 2016	https://doi.org/10.1038/nplants.2016.74
Rosa chinensis	Dicot PLAZA v5.0.0	Raymond et al., 2018	https://doi.org/10.1038/s41588-018-0110-3
Solanum lycopersicum	Dicot PLAZA v5.0.0	The tomato genome consorium, 2012	https://doi.org/10.1038/nature11119
