# Supplementary figures and images for: Genomic basis of scent loss and functional divergence in wild and cultivated carnations (Dianthus spp.)
Source: Hortic Res. 2026 Apr 7;13(8):uhag130. doi: 10.1093/hr/uhag130 (PMC13412721; doi:10.1093/hr/uhag130)

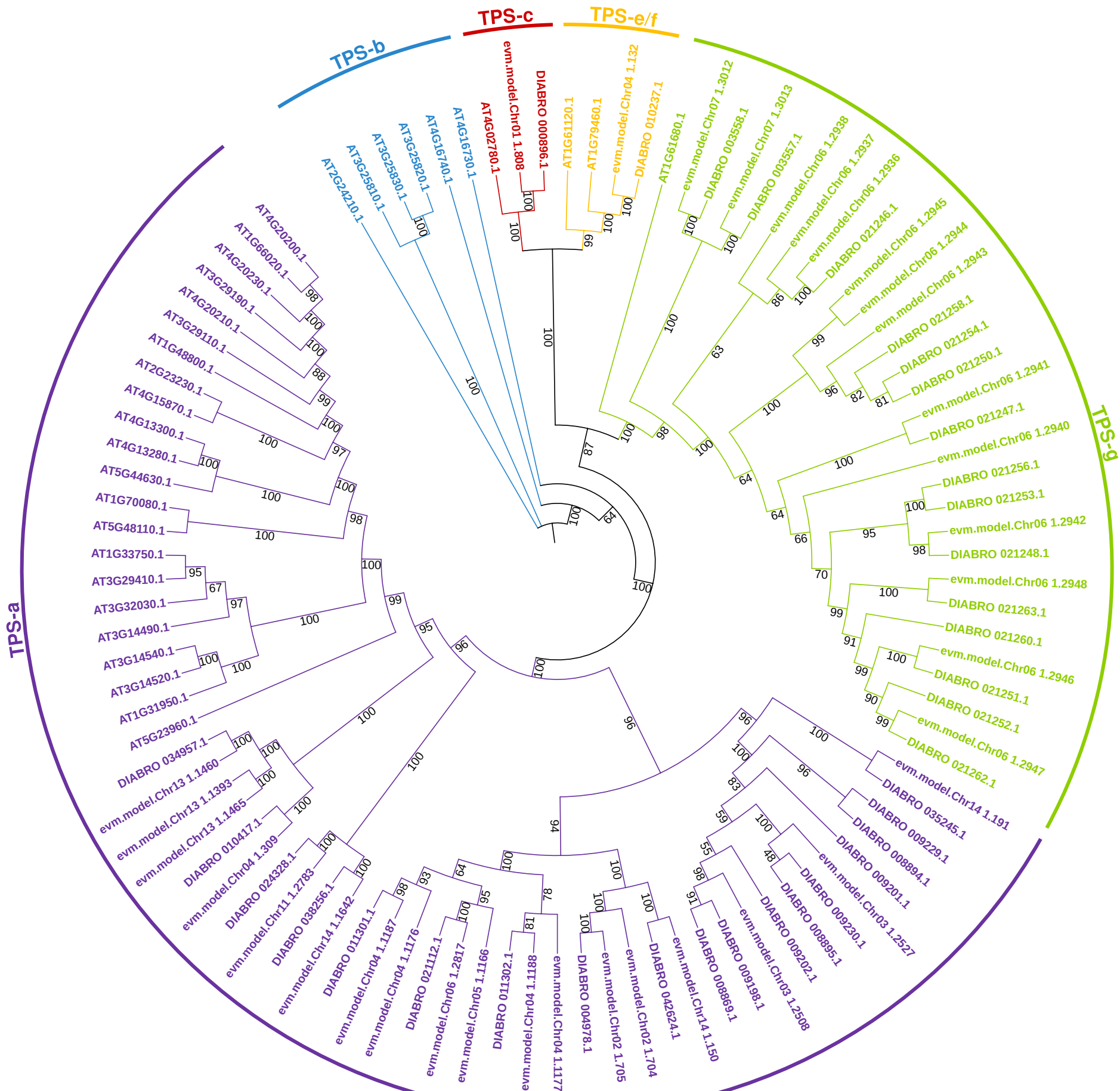

Supplement: Web_Material_uhag130 [file web_material_uhag130.zip › SUP_FIGURE1.pdf]

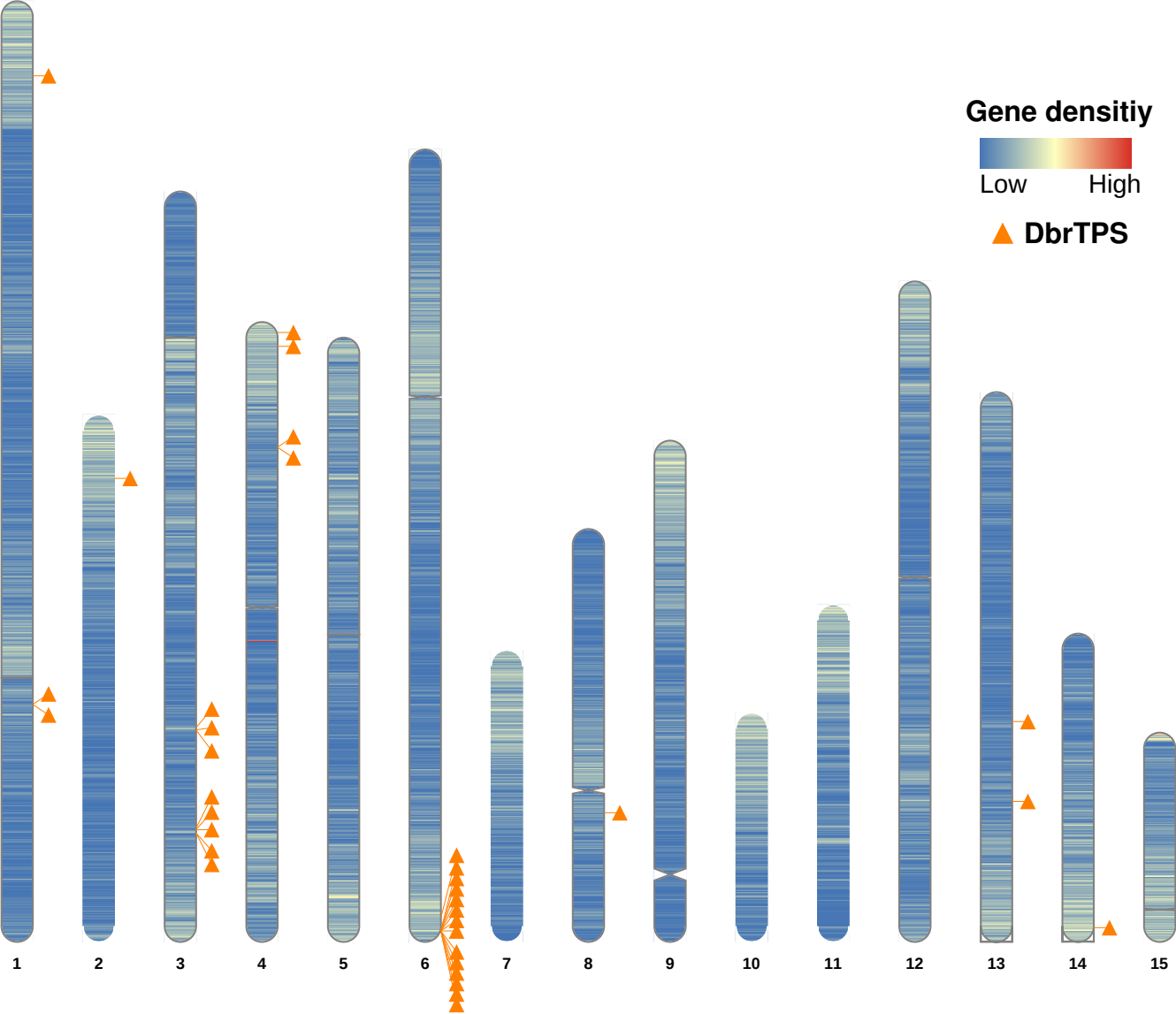

Supplement: Web_Material_uhag130 [file web_material_uhag130.zip › SUP_FIGURE2.pdf]

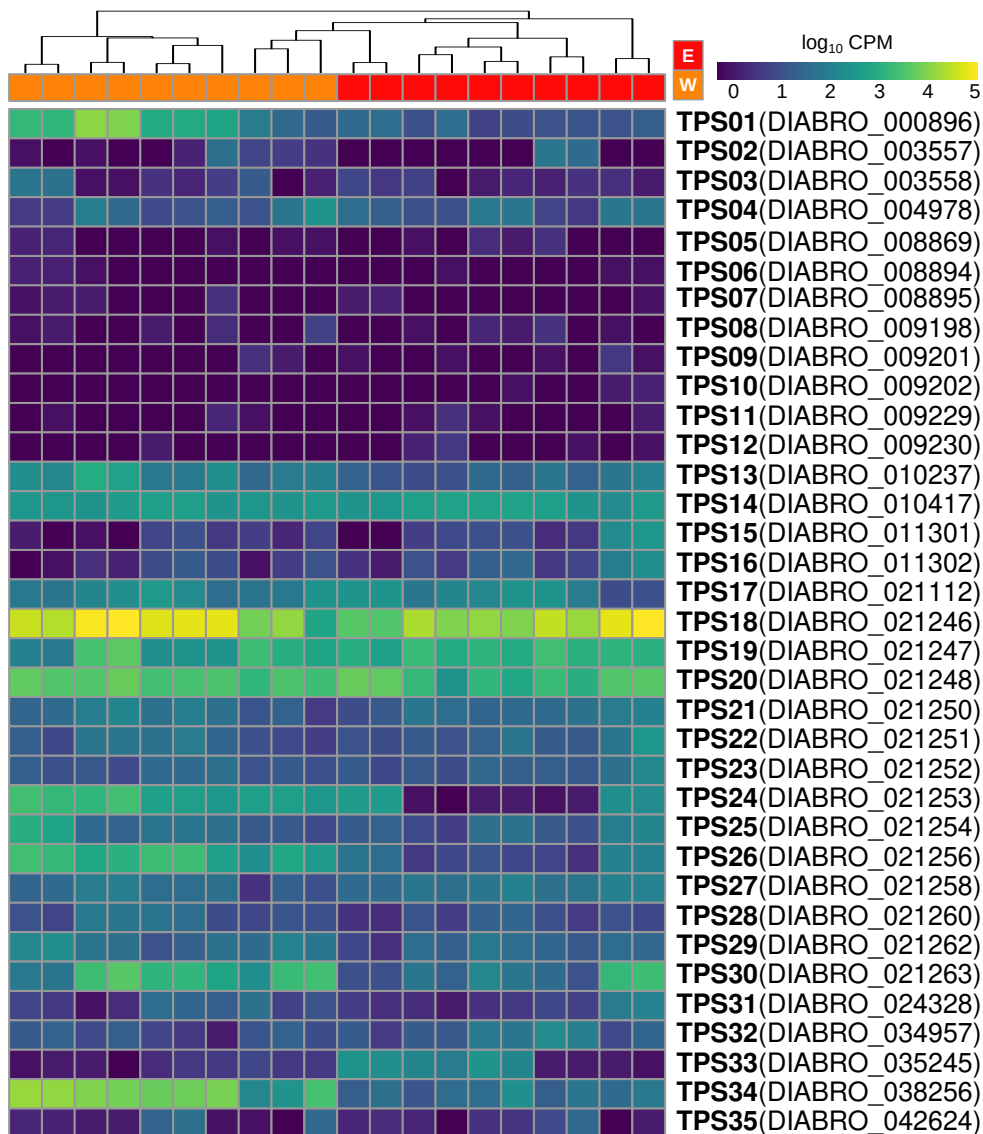

Supplement: Web_Material_uhag130 [file web_material_uhag130.zip › SUP_FIGURE3.pdf]

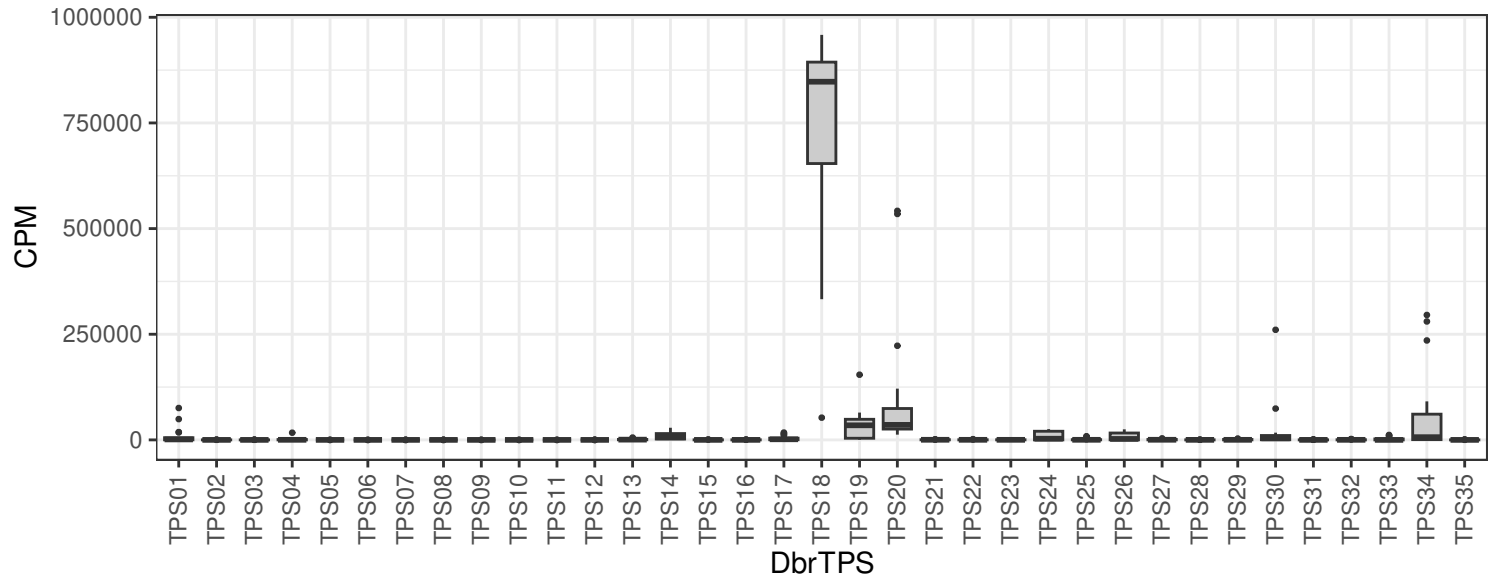

Supplement: Web_Material_uhag130 [file web_material_uhag130.zip › SUP_FIGURE4.pdf]
